# The Expansion of the Kensington Hospital

**Published:** 1911-06-17

**Authors:** 


### HOSPITALS OF TO-DAY.

### VIII.—THE EXPANSION OF THE KENSINGTON HOSPITAL.

Few people who pass by the present ugly front of the Kensington and Fulham General Hospital, have the least idea what lies behind it. Its chequered history from its foundation in 1887 to its removal and extensions on its present site, the newspaper controversies that at one time beset its path, the investigation by King Edward's Fund, its bankruptcy, and its subsequent resuscitation: here for its future historian is a brief career sufficiently crowded with incident!

Going in at the front door you find a private house which has been adapted to comprise a small ward, a nurses' sitting room, and, we believe, an office. The portrait of the pious founder hangs in the hall to remind all comers of the strange conditions under which the work was originated. Pass through the passage at the back of the original front and you enter the different world of a modern building which has been designed and not adapted for hospital work. This is the first extension to the old house, which in 1901 was used as an out-patient department, and which is now used as a ward, the consulting rooms which still abut from it being used as small rooms for paying patients. At the far end is the door into the dispensary which thus opens out of the ward, and is the architectural connection between it and the second extension which in 1905 gave a new out-patient department to the hospital. The hospital is thus a long narrow building with no storeys, on one side lying parallel to a road and on the other possessing a narrow strip of disused grassland which one day no doubt will be brought into cultivation as a garden or covered with buildings. As the site is a corner one and all the new buildings lie along the side street the old front successfully conceals all the progressive work that has been done, and remains the sole monument of the difficulties of the past. A new front is therefore considered necessary, and this not merely for decorative reasons but in order that the patients now housed in such inadequate quarters may be placed in properly designed wards, and that the nurses and domestic staff may be properly accommodated in the hospital building. Further, in this it is hoped to include a small children's ward, so that the original intention of the hospital to provide a few beds for them may be carried out.

It is most important to emphasise at this point that the Coronation appeal for £5,000 to re-front the hospital is an appeal for reconstruction and not for extension. The function of the Kensington and Fulham General Hospital is peculiar. It lies, as will be remembered, between St. George's and the West London, and its object is to provide a small hospital with a fixed number of beds for its section of West London and to serve with its out-patient department three boroughs. When the King's Fund examined the position of affairs it laid down this principle, that an efficient small hospital, with a good out-patient department and with a limited number of beds, was wanted for Fulham and Chelsea.

Reference has been made to the out-patient department. In April 1907 there were 4,700 out-patients on an average treated every month. In May a suggestion was made, we believe, by Mr. Louis McCausland, the secretary, that some charge, however small, should be exacted, except in those cases where it would clearly be impossible. This suggestion was adopted and in two months the numbers had fallen from 4,700 to 2,100. To-day the pennies collected, in most cases threepence a week, produce £250 a year. The result has been that the annual subscriptions have increased and multiplied, and in time, it is hoped, will form the staple of the hospital's income.

The hospital is busy collecting money and providing the stimulus of a variety of funds to those in the neighbourhood. There is the maintenance bed fund, to which the patient, especially the non-wage earning wife or mother, induces her husband to subscribe; there is the threepenny brick system which is assisting in the collection of the building fund, and there is the million shilling fund. The recently appointed president, Lord Claud Hamilton, as The Hospital noted on April 22, is busy in the final resuscitation of the institution. One of his encouragements is the grant from the Hospital Sunday Fund. Another energetic worker is Lieut.-Colonel H. J. F. Praeger, the chairman of the board, who is in the hospital or receives a visit from the secretary almost every day in the year. Naturally the present moment is full of strenuous endeavour for the staff. On Tuesday last the Coronation Festival dinner was held under the presidency of Lord Claud Hamilton, and some two hundred supporters of the hospital assembled. In an amusing speech the chairman described the position of affairs, and was followed by Mr. Hayes Fisher. We understand that some £400 was announced in subscriptions. No appeal was made at the dinner itself. The tables were decorated with gladioli and smilax and the most artistic song was "Spring is Coming," by Miss Cowling.

